# The use and profitability of hermetic technologies for grain storage among smallholder farmers in eastern Kenya

**DOI:** 10.1016/j.jspr.2020.101618

**Published:** 2020-05

**Authors:** D. Baributsa, A.W. Njoroge

**Affiliations:** Department of Entomology, Purdue University, 901 W. State Street, W. Lafayette, IN, 47907, USA

**Keywords:** Postharvest, Grain losses, Chemical-free storage, Technology scale-up, Return on investment

## Abstract

Hermetic storage technologies (HSTs) have been disseminated in Sub-Saharan Africa (including Kenya) to reduce grain storage losses among farmers. We carried out a study in three counties in eastern Kenya to assess the use and profitability of HSTs among farmers. Data were collected from 613 farmers using a semi-structured questionnaire and Kobo Toolbox via android tablets. Results showed an increase in use of HSTs among farmers from 53.7% in 2015 to 91.2% in 2017. PICS was the most used hermetic bags by farmers (84%) in 2017. Majority of farmers (73.5%) received training in the use of HSTs from extension agents and agro-dealers. About 40% of respondents purchased additional (one to five) bags after their first experience using them. The quantity of grain produced made up about half of the farmer’s decision to store. The primary reason (87%) farmers used hermetic bags was the need to manage insect pests. Maize and beans were the most produced and most stored crops; but maize was the most stored in HST. Grain price seasonality showed a near doubling effect between the lean and harvest seasons. Estimates of the return on investments (ROI) ranged between 13 and 80% for all crops and maize stored in hermetic bags had the highest ROI. Awareness and trainings are key in increasing adoption and proper use of HSTs.

## Highlights

• Use of hermetic storage technologies (HSTs) in Eastern Kenya significantly increased from 2015 to 2017.
• PICS bags were the leading brand but AgroZ bags’ market share was increasing.
• Maize was the most stored crop in HSTs followed by common beans and pigeon peas.
• Maize was the most profitable crop (highest return on investment) to store in hermetic bags.

## 1 Introduction

Farmers face many post-production constraints (Jayne et al., 2010). Maize postharvest losses resulting from insect or rodent damage and microbial contamination can reach 14–36% (Tefera, 2012). Access to storage technologies is limited due to high costs, credit constraints, lack of information, unavailability of good products, and risk aversion (Kadjo et al., 2013; Midega et al., 2016). Lacking good storage technology, many farmers sell immediately after harvest to avoid storage pest losses and therefore forgo potential profits (Stephens and Barrett, 2010; Barrett et al., 2010). For pest management strategies, some farmers resort to conventional insecticides and traditional grain protectants that have had limited success in preventing storage losses. In addition, the use of insecticides often results in misuse and overuse leading to food safety issues as well as insect resistance (Fields, 2006; Talukder, 2009).

In Kenya, grain storage at the household level is a basic necessity for ensuring food security. Farmers use different grain storage methods ranging from traditional granaries to insecticides. Many of these storage methods have limitations including cost, use, effectiveness, scalability, and safety. To preserve their harvested grain, farmers need technologies that are cost-effective, user-friendly, affordable and safe. The development of improved hermetic storage technologies (HSTs) i.e., hermetic bags and silo technologies, provide an alternative to conventional storage methods such as pesticides. In Kenya, hermetic metal silos were introduced by the International Maize and Wheat Improvement Center (CIMMYT) in 2009 to reduce postharvest storage losses of maize (Kimenju and De Groote, 2010). In 2013, Purdue Improved Crop Storage (PICS) bags were introduced to farmers in Kenyan by Purdue University (PFI, 2013). The introduction of PICS in Kenya was based on research findings that showed the effectiveness of PICS bags for storing various cereal and legume crops (Baoua et al., 2014; Njoroge et al., 2014; Ng’ang’a et al., 2016). Currently, five different hermetic storage bags have been certified for commercialization in Kenya including: Purdue Improved Crop Storage (PICS) bags, ZeroFly® Storage Bags, Elite Storage Bags, SuperGrainbags™ and AgroZ Storage Bags (KAAA, 2016).

All these HSTs take pride in preventing insect infestation, stopping aflatoxin development, and preserving seed quality without the use of insecticides (Murdock et al., 2012; Williams et al., 2014; Coffi et al., 2016; Likhayo et al., 2018). These technologies were introduced at an opportune time when Kenyan farmers were battling several storage pests including the larger grain borer *Prostephanus truncatus* (Horn) (Coleoptera: Bostrichidae) and *Sitophilus zeamais* (Motschulsky) (Coleoptera: Curculionidae), the most important pests on maize in the country (Mutambuki and Ngatia, 2012). To further assess adoption of hermetic storage in Kenya, on-farm and on-station trials compared the effectiveness of metal silos, super grain bags and polypropylene bags with or without Actellic super dust to control *S. zeamais* and *P. truncatus* on artificially infested maize (De Groote et al., 2013; Likhayo et al., 2016; Mutambuki et al., 2019). All hermetic technologies were effective in preventing insect infestation. A randomized controlled trial (RCT) monitored for a period of six months established that hermetic bags were profitable when used by farmers to store grain for at least four months, and if the bags were reused for at least four seasons (Ndegwa et al., 2016).

To increase awareness and adoption of hermetic technologies several efforts have been implemented by various projects. In 2013, 2014, demonstrations on PICS bags were implemented in 850 villages and farmers’ groups, and markets in the Rift Valley, and Central and Eastern regions of Kenya through a grant to Purdue University by Feed the Future Partnering for Innovation (PFI, 2013). In addition, the Kenya Agricultural Value Chains Enterprises Project (KAVES) promoted PICS bags in 2013–2016 (Foy and Wafula, 2016). Bell Industries, the licensed manufacturer and distributor of PICS bags sold more than 900,000 PICS bags (100-kg storage capacity) from 2013 to 2016 (Foy and Wafula, 2016). The early success of PICS bags in demonstrating the effectiveness of hermetic storage in Kenya resulted in competition – private companies such as GrainPro in the U.S and A to Z in Tanzania became interested in selling (or expanding their markets) hermetic technologies to farmers in Kenya. In an effort to scale-up the adoption of the all hermetic technologies by encouraging competition, KAVES and the Government of Kenya organized a national campaign in November 2016 (FtF, 2017). Hermetic bags including PICS, AgroZ, SuperGrainbags™, Zerofly®, and Elite bags were promoted during these national campaigns (road shows and media advertisements).

Further, in 2014, an international consortium (a partnership between Australia, Canada, the United Kingdom, the United States, the World Bank, and the Bill and Melinda Gates Foundation) initiated the AgResults program in Kenya that used a Pay-for-Results prize competition to motivate private sector competitors to commercialize HSTs (plastic bags, plastic tanks, and metal silos) to smallholder farmers (AgResults, 2019). Hermetic bags sold under the AgResults program included PICS, AgroZ, SuperGrainbags™, Zerofly®, and Elite bags (AgResults, 2018a). The composition and size of these bags varied depending on the manufacturer: single, double and triple layer bags; and 50, 90, 100 and 250 kg capacity. Some of these bags were locally or regionally manufactured (PICS, AgroZ and Elite), and other imported from outside Africa (SuperGrainbags™ and ZeroFly®). As a result of this aggressive marketing, about 1.4 million devices were sold between 2015 and 2018. This represented an equivalent of 413,265 metric tons of improved storage capacity at the farmers’ household level (AgResults, 2018b).

Despite these studies and interventions to increase awareness and adoption of hermetic technologies in Kenya, there is limited information about how farmers were accessing and using these innovations. Accordingly, the present study was conducted to assess the (i) use of hermetic storage technologies at the farm-level after several efforts to build awareness; (ii) access to information and training on hermetic storage technologies; and (iii) availability and profitability of hermetic bags for grain storage.

## 2 Materials and methods

The data collected for the current study were part of a larger survey conducted to understand farmers’ postharvest management practices of grain in eastern Kenya. Full details of material and methods can be found in Njoroge et al. (2019). Below are the key elements of the material and methods relevant for this study.

### 2.1 Study area

**Fig. 1:**
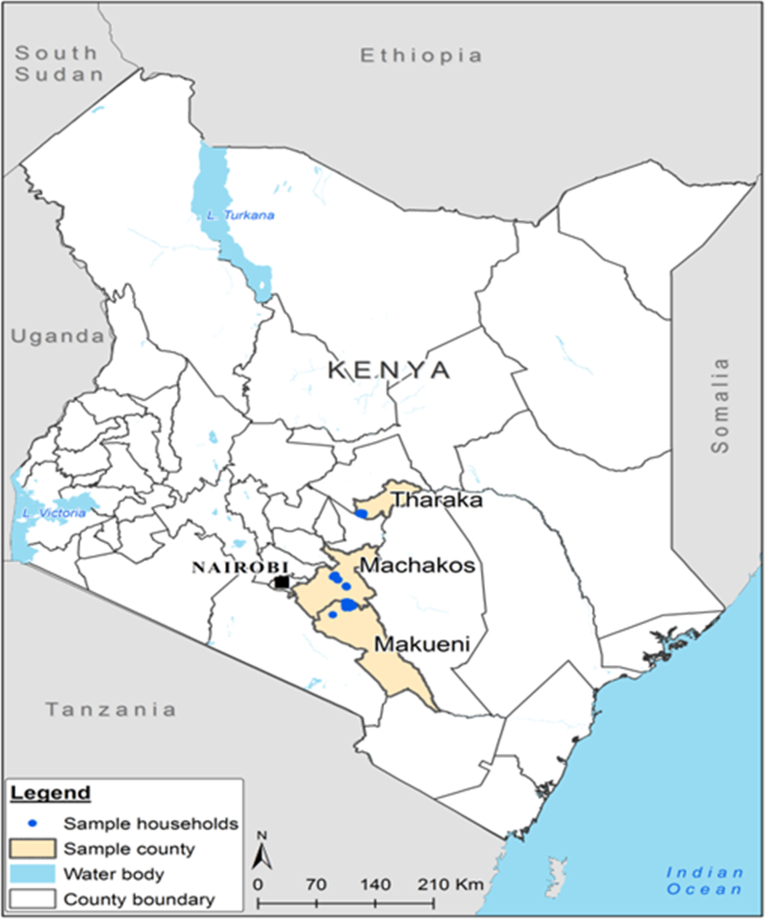
Map of Kenya showing the study sites (in blue dots) in three counties in Eastern Kenya Source: Data collected during the study and mapped using QGIS software.

This study was conducted in October/November 2017 in Tharaka Nithi, Makueni and Machakos counties located in Eastern Kenya (Fig. 1). These counties represent important cereal and legume grain production areas where hermetic storage bags (PICS, AgroZ, SuperGrainbags™, Zerofly®, and Elite bags) were disseminated. These areas benefited from awareness building and training in the use of HSTs by several projects. The survey targeted farmers in areas with high use of HSTs. Villages where the survey was conducted were identified with the help of local extension agents. The geographic limitations of the study were the result of financial and logistical constraints.

### 2.2 Sampling, data collection, and analysis

We interviewed farmers in 50 villages in three counties (16 villages in Tharaka-Nithi, 17 villages in Makueni, and 17 villages in Machakos). Thirteen respondents were interviewed in each village (a total of 613 farmers). Of the 613 sampled farmers, 559 were HST users and 54 were non-users. Respondents were randomly selected from a list of farmers in a village located in areas where hermetic bags had been promoted. A semi-structured questionnaire was used to collect information from the respondents. Data collected included storage practices, access to information, hermetic storage use and management, availability of HSTs, and price of grains at harvest and during the lean seasons. The questionnaire was designed with and transferred to Kobo Toolbox. Data were collected using Kobo Collect app uploaded to android tablets. The data were saved on the Kobo server for storage and visualization. Later it was downloaded as excel sheets for further analysis. Quantitative and qualitative data collected were coded and analyzed using the Statistical Package for Social Sciences (IBM Corp, 2016; New York, NY, United States). Descriptive statistics, means and cross-tabulation tables were done to summarize the key findings of the study. Pearson correlation was done to assess associations among variables.

## 3 Results

Data on demographic, socio-economic and farming system characteristics were published in Njoroge et al. (2019). The majority of respondents were female (64%), were married (87%), had 10 years or more of farming experience (82%) and had more than a high school education (57%). The average size of a household was 5 people.

### 3.1 Access to extension services, information, and training on hermetic storage technologies

Table 1 illustrates access to information and training on HSTs. About 52.7% of the respondents did not have contact with extension services. Among those who had contact with extension services, 19.4% had links to government research/extension services, 15.3% were connected to farmers’ groups and only 11.4% had contacts with Non-governmental organizations (NGOs) and projects. The largest number of respondents received farming information from other farmers/neighbors (48.1%) and from researchers/extension agents (25.4%). Most respondents (99.5%) were aware of HSTs (data not shown). Seventy-five percent (75%) of respondents first heard of HSTs in 2015 and 2016, while 22% first heard of hermetic bags in 2013 and 2014 (data not shown). Respondents received information on HSTs from several sources: other farmers (49.9%) and radio (47.8%), extension agents (28.2%) and agro-dealers (27.1%). About 79.1% of farmers had received training in the use of HSTs in 2015 and 2016. Training was mainly provided by extension officers (37.8%) and agro-dealers (35.7%).

**Table 1:** Contact with extension services, source and access to hermetic storage technologies (HSTs) information, and training in HSTs in three counties of Eastern Kenya.

	Variable	% Respondents
	Government research and extension	19.4
*Contact with extension services n* = *613*	NGOs/project extension	11.4
	Farmer group extension	15.3
	Other sources	1.1
	No access to extension	52.7
*Source of extension information n* = *613*	Self/neighbors/other farmers	48.1
	Extension/research agents	25.4
	Media	9.3
	NGO projects	9.1
	Input dealers	2.4
	Other sources	5.6
*Source of information on HSTs* n = 559	Other farmers	49.9
	Radio	47.8
	Extension agents	28.2
	Agro-dealers	27.1
	TV	3.1
	Roadshow	2.4
	Newspaper	0.2
	Leaflets/pamphlets/brochures	1.0
*Year received HST training* n = 426	2013	4.0
	2014	14.6
	2015	45.3
	2016	33.8
	2017	2.3
*How received HST training* n = 426	Agro-dealers	35.7
	Extension officers/village demonstrations	37.8
	Farmers/neighbors	12.0
	Other sources	8.2
	Training posters	1.9
	Market demonstration	3.3
	Road show	1.2

### 3.2 Sourcing and reasons for using hermetic storage technologies

Each of the study sites had shopping centers close to the farmers’ homes. The distance to the nearest markets varied from less than half a kilometer to 5 km for about 94.1% of respondents (Table 2). About 79.9% of respondents could purchase HSTs in shops and markets within 5 km from their houses. The proportion of farmers who used HSTs was 91.2% among whom 87.1% adopted the technology for insect pest management (Table 3). About 8.8% (54 out of the 613 respondents) did not use HSTs. Reasons for not using HSTs (among the 54 respondents) included high price (46.3%), lack of knowledge on how to use the technologies (22.2%), and unavailability (14.8%) (Table 3). About 23% of respondents noted that unavailability of hermetic technologies at harvest was a major constraint in Machakos county. In addition, among users of HSTs, 96.2% bought the bags while 3.8% received them for free. Eighty percent (80%) of respondents bought the bags from vendors (Table 3). The majority (82.5%) of respondents noted that retail points such as shops and markets are the most convenient locations to make HSTs available to farmers (Table 3). After exposure to HSTs, the majority of farmers (80.5%) bought between 1 and 5 hermetic bags the first time (Table 2). Among the 43.1% who bought the bags the second time, 66.4% purchased 1–5 bags (Table 2).

**Table 2:** Distance from farmers’ house to nearest market and to where hermetic storage technologies (HSTs) are sold, and number of bags purchased in three counties in Eastern Kenya.

Variable	% Respondents	
Distance	To nearest market (N = 559)	To nearest place to buy HSTs (n = 559)
<0.5 km	18.2	18.4
0.5–1 km	28.9	23.7
1–5 km	47.0	37.8
5–10 km	5.9	13.9
>10 km	0	6.2
***Number HST bags bought***	***First purchase (n = 559)***	***Second purchase (n = 243)***
1–2	43.6	31.5
3–5	36.9	34.9
6–10	12.7	19.8
11–20	4.5	10.3
More than 20	2.3	3.2

**Table 3:** Reasons for and for not using hermetic storage technologies (HSTs), constraints among users, acquisition, sourcing and most convenient way to make HST’s available in three counties in Eastern Kenya.

	Variable	% Respondents
*Reasons for using HSTs n* = *559*	Insect management	87.1
	Mold/aflatoxin management	2.9
	Rodent management	2.4
	Others	7.6
*Reasons for not using HSTs n* = *54*	Expensive	46.3
	Insecticides are cheaper	13.0
	Unavailable	14.8
	Don’t know how to use	22.2
	Don’t know where to buy	3.7
*Issues among HSTs users* n = 559	Reduced grain quality	13.5
	Not available at harvest	13.2
	Poor drying/storage facilities	13.4
	Lack of training on their use	10.9
	Unfavourable prices after storage	2.3
	No issues	66.2
*Acquisition of HSTs n* = *559*	Buy	96.2
	Receive for free	3.8
*Source of HSTs n* = *559*	Vendors	80.0
	Farmer organizations	7.2
	NGOs	5.0
	Other sources	4.8
	Projects	2.5
	Government	1.1
*Most convenient way to make HSTs available n* = *559*	Shops	65.4
	Markets	17.1
	Grain traders	4.6
	Extension workers	10.6
	Farmer based organizations	1.3
	Others	1.0

### 3.3 Knowledge, use, and handling of hermetic technologies during grain storage

Knowledge about the different HSTs varied among the respondents: 84.8% for PICS, 24.6% for AgroZ, and 3.9% for the other technologies (ZeroFly®, SuperGrainbags™, Elite, Metal Silos) (data not shown). Overall, hermetic bags use among farmers grew over a period of three years from 53.7% in 2015 to 91.2% in 2017 (Table 4). In 2017, farmers mainly used PICS bags (84.0%) followed by AgroZ (11.4%). Farmers who used Elite, SuperGrainbags™, Zero Fly and Metal silos accounted for only 3.1% of the respondents. Trends in use of different HSTs over the three years showed an increase in adoption: PICS 48.7% in 2015 to 79.1% in 2017; Agro Z 7.8% in 2015 to 11.4% in 2017 (Table 4). It is worth noting that the number of farmers not using HSTs decreased over the years (from 46.3% in 2015 to 8.8% in 2017) and those using more than one technology increased during the same time period (from 0.82% in 2015 to 3.6% in 2017) (data not shown).

**Table 4:** Change in the use of hermetic storage technologies (HSTs) by farmers from 2015 to 2017 in three counties in eastern Kenya.

Hermetic storage technology	2015 (n = 613)		2016 (n = 613)		2017 (n = 613)	
	Frequency	%	Frequency	%	Frequency	%
Overall use of HSTs	329^a^	53.7	538	87.8	559	91.2
PICS	300	89.8	474	85.6	488	84.0
SuperGrainbags™	1	0.3	3	0.5	3	0.5
ZeroFly®	1	0.3	2	0.4	4	0.7
AgroZ	26	7.8	62	11.2	66	11.4
Elite	2	0.6	6	1.1	8	1.4
Metal silos	0	0.0	0	0.0	3	0.5
Others^b^	4	1.2	7	1.3	9	1.5

a The number of users of HSTs is lower than the total users of each individual technology because some farmers were using more than one technology.

b Others refer to unbranded hermetic bags as well as traditional hermetic containers such as jerricans, pots and gourds.

Major factors that motivated farmers to store grain using HSTs included (i) the quantity of grain produced (51.5%), (ii) availability of credit to buy hermetic bags (24%), and (iii) low grain prices at harvest (26%) (Table 5). There was a strong correlation between the quantity of grain produced and stored: 84% for maize, 77.4% for beans, and 84.4% for pigeon pea (data not shown). Farmers reported that they would likely reuse their HSTs for several years – 88.5% of the respondents said they would likely reuse their bags for 2–3 or more seasons (Table 5). Half of farmers (50.4%) opened their bags on a monthly basis (Table 5). About 54.3% of respondents noted that the main reason for opening the bags was to withdraw grain for home consumption, while 33.7% said it was for both consumption and sale. After withdrawing grain, the majority of farmers (85.6%) left the bags opened for less than 30 min. Only a few closed the hermetic containers immediately after opening (7.2%). Though about a third of respondents had issues with rodents and insects, 68.2% had no challenges. About 22% of farmers reported rodent attacks; while 8.8% of respondents complained about insects making holes on liners from inside and outside the bags. Most farmers (85.5%) had no issues with quality of hermetic technologies being sold on the market (Table 5). Quality issues were reported by some farmers and they included: defective bags (8.2%), liners that cannot be reused (2.4%) and counterfeit technologies (4.4%).

**Table 5:** Summary of hermetic storage technologies (HSTs) use and practices in three counties in eastern Kenya.

	Variable	% Respondents
*Decision to store grain in HSTs* (n = 559)	Quantity produced	51.5
	Low prices at harvest	10.4
	Immediate needs	4.4
	Credit to buy bags	24.1
	None	9.5
*Reuse of HSTs* n = 559	No reuse	4.1
	1 season	7.3
	2 seasons	8.6
	3 seasons or more	79.9
*HSTs Opening frequency* n = 559	Monthly	50.4
	After 2 months	6.4
	After 3 months	15.3
	More frequently	21.4
	Never open	6.5
*Duration farmers leave bags open* n = 523	<30min	85.6
	30–60min	2.9
	>60min	4.2
	Never leave open	7.2
*Reason for opening* n = 559	Consumption	54.3
	Sale	5.7
	Both	33.7
	Others	8.6
*Challenges during hermetic storage* n = 559	Rodents	22.0
	Insect damaging grain	12.6
	Insects making holes on liners from inside the bags	5.7
	Insect making holes on liners from outside the bags	3.1
	No challenges	68.2
*Quality issues with HSTs* n = 559	Defective	8.2
	Counterfeit	4.4
	Cannot be reused	2.4
	Susceptible to insect damage	6.9
	No quality issues	85.5

### 3.4 Main crops produced and stored, and their profitability in hermetic storage technologies

The main crops produced were maize (98% of farmers surveyed), beans (66%) and pigeon peas (28%) (Njoroge et al., 2019). Additional crops produced included green gram, and cowpea. Among the respondents, 90% produced and 75% stored 270 kg (3 bags) or more of maize while only 45.4% produced and 23% stored 270 kg or more of beans (Table 6). Farmers who produced and stored 6 or more bags were: 64% and 40% for maize, respectively; 14.6% and 6.5% for beans, respectively; and 2.6% and 1.4% for pigeon peas, respectively. The majority of respondents producing legumes stored small quantities (2 bags or less) of beans (76.8%) and pigeon peas (93.3%). Storage in HSTs varied by crops and quantity stored. Respondents who used 3 hermetic bags or more were 47.6% for maize, 16.8 for beans and 18.4% for pigeon peas (Table 6). There were significant correlations (*p* < 0.01) between quantity produced and stored: 84% for maize, 77.4% for beans and 84.4% for pigeon peas (data not shown). Significant correlations (*p* < 0.01) were also observed between quantity of grain stored and storage in HSTs: 24% for maize, 46.2% for beans and 46.6% for pigeon peas (data not shown). Price seasonality (price at harvest and during the lean season) varied among crops and sometimes between the different counties (Table 7). Estimates of the return on investments (ROI) varied between crops and counties: 61–80% for maize, 50–59% for beans, 43–59% for pigeon peas, 13–63% for cowpea and 30–38% for mung beans.

**Table 6:** Quantity of grain produced, total grain stored and grain stored in HSTs (90 kg-bags) by farmers (%) in three counties in eastern Kenya.

Variable	Maize∗			Beans∗			Pigeon peas∗		
Quantity (90-kg bags)	Produced	Total stored	Stored in HST	Produced	Total stored	Stored in HST	Produced	Total stored	Stored in HST
	*n* = *613*	*n* = *613*	*n* = *559*	*n* = *613*	*n* = *613*	*n* = *320*	*n* = *613*	*n* = *613*	*n* = *163*
Less than 1	4.6	12.6	17.3	29.4	53.0	29.4	72.1	83.5	39.9
1–2	5.4	12.4	35.1	25.1	23.8	53.8	13.7	9.8	41.7
3–5	25.4	34.9	24.4	30.8	16.5	10.6	11.6	5.2	12.3
6–10	27.9	23.6	11.8	10.3	4.1	2.2	1.6	1.1	4.9
11–20	21.0	11.6	6.2	3.6	2.4	.9	0.8	0.3	1.2
More than 20	15.7	4.9	5.2	0.7	–	3.1	0.2	–	–

**Table 7:** Estimates of the return on investment (ROI) when farmers store grain for 6 months using 100 kg hermetic storage bags in Machakos, Makueni and Meru counties in eastern Kenya. Grain and HST prices are in Kenya Shilling (KES). KES 250 is the recommended retail price of PICS bags.

		Price (KES/90 kg bag)		KES				Percent
County	Crop	Harvest	Lean season	Gross margin	Price HSTs∗	OCC∗∗	Net Gain	ROI∗∗
Machakos	Maize (n = 226)	2143.90	4500.00	2356.10	250.00	215.45	1890.65	78.98
	Beans (n = 158)	4066.50	6869.00	2802.50	250.00	388.49	2164.02	50.13
	Pigeon Pea (n = 141)	3817.70	6820.00	3002.30	250.00	366.09	2386.21	58.66
	Cowpea (n = 63)	3561.90	6557.10	2995.20	250.00	343.07	2402.13	63.02
	Green Grams (n = 52)	4996.20	7713.50	2717.30	250.00	472.16	1995.14	38.03
Makueni	Maize (n = 177)	1916.90	4104.70	2187.80	250.00	195.02	1742.78	80.43
	Beans (n = 203)	3780.50	6784.40	3003.90	250.00	362.75	2391.16	59.33
	Pigeon Pea (n = 152)	3513.50	6010.90	2497.40	250.00	338.72	1908.69	50.72
	Cowpea (n = 26)	3303.80	5688.50	2384.70	250.00	319.84	1814.86	51.07
	Green Gram (n = 13)	6192.30	9000.00	2807.70	250.00	579.81	1977.89	30.70
Tharaka Nithi	Maize (n = 170)	2418.50	4541.20	2122.70	250.00	240.17	1632.54	61.18
	Beans (n = 163)	4191.70	7345.10	3153.40	250.00	399.75	2503.65	56.37
	Pigeon pea (n = 5)	5000.00	8000.00	3000.00	250.00	472.50	2277.50	43.38
	Cowpea (n = 14)	7178.60	9071.40	1892.80	250.00	668.57	974.23	13.11

## 4 Discussion

### 4.1 Access to extension services, information, and training on hermetic storage technologies

About half of the farmers did not have access to extension services. Those with access relied on several sources of agricultural information including government research and extension services, NGOs, and other farmers. The limitation in access to extension services is related to weak government support to these farmers. A study in Kenya showed that farmers growing low-value crops with limited surplus to market and living in remote areas are poorly served by extension services (Muyanga and Jayne, 2006). Given the limited access to extension services, the majority of farmers relied on other farmers as their source of information on general agriculture and HSTs. This shows the importance of reaching farmers directly with information on agriculture or new technologies and innovations. Word-of-mouth plays an important role in disseminating information on HSTs.

Development efforts to promote postharvest solutions to reduce storage losses at the farm-level ensured that almost all farmers were aware of HSTs. The findings of this study and other studies have shown that insect pests are the major challenge during grain storage in Sub-Saharan Africa (Abdoulaye et al., 2016). PICS technology was the first hermetic bags to be scaled-up in Kenya. In 2013, 2014, PICS reached some farmers in the survey area. This explains the low number of farmers who were aware of HSTs in both years. A study conducted in West and Central Africa in 2012 showed that lack of information was the second most important reason farmers were not using hermetic storage bags (Moussa et al., 2014). In 2015, 2016, increasing numbers of development partners such as the USAID KAVES project, NGOs, and private sector actors (agro-dealers) contributed to increased awareness. These efforts explain the higher number of awareness and trainings on HSTs in both years. Farmers who participated in postharvest trainings facilitated the spread of HST information. Training increases knowledge on the attributes and the use of HSTs, and provides information on sourcing (availability) of the new technologies in the market. Though the majority of farmers attended trainings that may have included free HSTs samples for demonstrations, nearly all of them purchased the bags. Extension services and agro-dealers facilitated trainings or other extension activities. Several studies have shown that farmers who live in villages where HST training activities have taken place are more likely to adopt the technology and share information with other farmers (Doss et al., 2003; Moussa et al., 2009, 2014). The higher number of women respondents in this survey is an indication that they need to be targeted with information and training on the use of hermetic bags. Women play an important role in postharvest management of grains (Moussa et al., 2009; Ibro et al., 2014; Moussa et al., 2014).

### 4.2 Sourcing and reasons for using hermetic storage technologies

Despite having markets within a 5 km radius, some farmers had to walk greater distances to find HSTs retail points. Our data shows that 94.1% of respondents had markets within 5 km, and about 80% of the respondents could buy HSTs within the same distance. In addition, while no farmer said the nearest market was 10 km away, more than 6.2% of respondents had to travel more than 10 km to purchase HSTs. Therefore, more efforts are needed to bring the hermetic technologies closer to the farmers. Studies have shown that adoption of HSTs such as the PICS bags by farmers is constrained by the unavailability of the technology in communities or rural markets (Moussa et al., 2014; Nouhoheflin et al., 2017). A study conducted in Niger and Burkina Faso showed that adoption of PICS bags significantly dropped if farmers had to travel more than 7 km from their homes to purchase the technology (Moussa et al., 2010). HST adoption among farmers can be enhanced by increasing the number of shops and retail points (markets) in rural areas that sell these technologies. Timely availability of HSTs in rural markets and shops is crucial. Farmers in remote areas need storage solutions at harvest but often complain that HSTs are available late (several weeks after harvest). In this study, some farmers cited unavailability as the key reason for not using HSTs. Non-users reported that the most important constraint for adoption was the high cost of HSTs. Retail price for most hermetic bags in Kenya is 250 KES per unit. Adoption studies conducted in areas where farmers were using HSTs in Sub-Saharan Africa have found that local unavailability and high prices are among the most common cited reasons for not using hermetic bags (Moussa et al., 2014). Improving awareness of HSTs among non-users would increase their willingness to pay for these technologies (Channa et al., 2019) as about half of those who complained about price had limited knowledge of the use of HSTs.

To sustain HST markets, agro dealers selling these technologies need to reach out to thousands of farmers through marketing and efficient distribution channels because most smallholder farmers buy a few bags (less than five bags the first time) (Foy and Wafula, 2016). There was an increase in the number of respondents (by 10%) who bought six hermetic bags or more during the second purchase. On average, the majority of buyers (61.1%) are farmers who purchased three hermetic bags to store grain destined for home consumption. The fact that more than four-fifths bought their bags from agro-dealers and within 5 km from their homes is an indication that the distribution system of hermetic bags is well developed in Eastern Kenya. In fact, most respondents knew where the bags were sold. This was the result of distributor-led demonstration events in rural areas that served the purpose of raising awareness among end-users after HSTs distributors rapidly identified hundreds of rural dealers who were interested in selling hermetic bags to farmers (Fintrac, 2016). Making HSTs available through vendors in agro-dealers’ shops and rural markets is crucial to increase adoption of hermetic bags.

### 4.3 Knowledge, use, and handling of hermetic technologies during grain storage

Studies conducted in Kenya show that the uptake of hermetic bags is higher in Eastern Kenya compared to the rest of the country (AgResults, 2018b). This study shows a steady increase in the use of hermetic technologies over the years in the three counties in Eastern Kenya. PICS as the first HST to be scaled in the Kenya market was better known by farmers than any other hermetic bags. The use of PICS bags increased over the three years more than any other hermetic technologies. Sales of PICS bags in Kenya grew from 3000 bags in 2013 to about 620,000 bags in 2016. This was due to aggressive marketing and advertising by donors and the PICS distributor that propelled the demand for this technology (Fintrac, 2016). Some of these organizations provided the PICS distributor with grants to build awareness through training and media activities. In addition, the PICS distributor received support as revolving funds to manufacture and develop the retail network that reaches farmers in rural markets (Foy and Wafula, 2016). PICS percent share of the market slightly decreased (though the number of users increased) over time to AgroZ due to its inclusion in the KAVES’ marketing roadshow. The small but growing number of farmers who were using more than one brand of hermetic bags was an indication of increased awareness of the different HSTs options for grain storage. The growing demand for HSTs by smallholder farmers has spurred entrepreneurs to develop new products that are either imitations or improvements of existing ones. The entrance of new products in the market is good for farmers as it provides more alternatives and increase competition among the different products. Competition may eventually lead to improved supply chains and result in lower costs for HSTs for farmers. However, more competition in the markets, especially from imitations, could result in the “race to the bottom” where companies are more worried about profits than providing good products to farmers. Such cases have been reported in Kenya leading the government to take an initiative for developing HST standards to deal with low quality materials that could pass as hermetic storage solutions (KEBS, 2019).

As hermetic bags are suited for farmers who produce enough to store, it is apparent that the quantity of grain produced influenced the decision to purchase HSTs. Farmers invest in hermetic bags because they are profitable and can be reused for several seasons. The finding of the present study corroborates that of Baributsa et al. (2014) who showed that most farmers reuse PICS bags to store grains for three or more seasons. Training has a positive impact on the management of HSTs during storage. Most farmers (71%) opened their bags once in one or several months in order to obtain grain for home consumption or sale. Once they remove grain, most of them did not leave the bags open for a long period of time. These management practices are really important in maintaining the hermetic conditions and preventing insect resurgence. In addition, training also helps in rodent management. Most HSTs, especially hermetic bags, are susceptible to rodent attacks. Despite a few major challenges during storage, most farmers take precautions in preventing rodent attacks including the use of cats and other rodent control measures such as rodent traps and rodenticides (Ndegwa et al., 2016). Field studies comparing HSTs have generally concluded that they are similar in effectiveness (Baoua et al., 2013; Coffi et al., 2016; Ndegwa et al., 2016). Farmers who store their grain in hermetic bags will need to decide based on several factors including cost, availability, and durability (reuse). Under certain circumstances, hermetic liners are holed by insects (De Groote et al., 2013; Baoua et al., 2013; Coffi et al., 2016). When hermetic bags with a single liner are damaged by insects, it limits their reusability. To address these issues, some manufacturers are impregnating HST liners and woven bags with insecticides. The question is whether these HSTs impregnated with insecticides can still be classified as chemical-free storage methods; which is the most important selling point for hermetic bags to farmers. Though metal silos are durable and less susceptible to pest damages (insects and rodents), their uptake has been low in Kenya due to limited dissemination and adoption among smallholder farmers. The major adoption challenge is the initial acquisition cost of metal silos (e.g. $35 USD/90 kg metal silo compared to $2.45 USD/100 kg hermetic bag) which is substantial for smallholder farmers given their limited resources (Kimenju and De Groote, 2010; Tefera et al., 2011; Walker et al., 2018). Other challenges associated with the use of metal silos and could affect adoption include their inability to maintain hermeticity around the seal due to manufacturing defects or damages during transport, and difficulties in removing the excess air when grain is not filled to the top or is removed over time leading (De Groote et al., 2013; Viola, 2017). Insect accessing oxygen during storage in metal silos could lead to the reinfestation of grain.

### 4.4 Main crop produced and stored in hermetic storage technologies, and their profitability

Farmers grew several crops – five legume and cereal crops - but the three major ones were maize, beans and pigeon peas. Maize was the first major crop produced and stored in hermetic technologies in all the surveyed areas. Maize is a major staple food crop in Kenya and is a source of income for farmers (Foy and Wafula, 2016; Ndegwa et al., 2016). Prior to the introduction of HSTs in Kenya, maize stored by farmers was devastated by the larger grain borer *P. truncatus* (30–90% losses) and the maize weevil *S. zeamais* (10–20% losses) (Mutambuki and Ngatia, 2012). The introduction of HSTs has helped to reduce maize losses to less than 1% (Njoroge et al., 2014). Common beans are also staple crops though some of the other legumes including green gram and pigeon peas are grown mostly by women as cash crops (Mergeai et al., 2001; Mboyah, 2018). Legume crops were produced in smaller quantities compared to maize as more than 85% and 97% of farmers produced less than 450 kg (5 bags) of beans and pigeon peas, respectively. Most of the grain produced was stored and later consumed or sold. Farmers who produce more grain were likely to store longer and use HSTs. More HST devices purchased were used to store maize than any other grains. The strong correlation that exists between grain produced and stored for all crops; and quantity stored and storage in HSTs is due to the role of maize, bean and pigeon peas in food security or in generating income for rural households. These results agree with findings that showed smallholder farmers typically consume a substantial part of what they produce and mainly store grain to ensure household food security (Sibhatu and Qaim, 2017). In addition, grain stored in HSTs is not treated with insecticides, which adds the benefit of feeding families and supplying consumers with chemical-free food. In fact, food safety (elimination of insecticide use) appears to be the major driver for smallholder farmers to use hermetic bags in Kenya (AgResults, 2019).

The seasonal change in grain prices varied for each crop and among crops in the three counties. Market prices of the different crops significantly increased from harvest time to the lean season. The differences in prices is explained by the increasing scarcity or surplus of grain in local markets during the months following harvest. Some crops were more profitable than others. For a minimal investment of 250 KES (US $2.45), farmers can double their income by storing grains in hermetic bags for several months. This is because the price of the different grains can double from harvest to the lean season (AgResults, 2018b). The Return on Investment (ROI) was higher for maize, suggesting that it is more profitable to store maize for sale than for any other crop. Maize is by far the most important food crop in Kenya; hence, there is always demand for grain in the market. Our findings corroborate other studies showing positive return for various crops in different countries. Funk et al. (2018) reported more than 80% maize price changes in Kenya from one year to the other. In West Africa, the ROI for cowpea ranged from 14 to 77% and 38–99% in Ghana and Mali, respectively (Baributsa et al., 2014). In Niger, Moussa et al. (2014) found that a farmer using a 100 kg PICS bag to store cowpea would make an additional cash flow of US $27. Building awareness among farmers of the benefits of HSTs in improving food security, income and health is important to increase adoption. Knowledge of the HSTs attributes increases the willingness of farmers to pay the suggested retail price for hermetic bags (Channa et al., 2019).

## 5 Conclusions

The use of hermetic storage technologies at the farm-level in Kenya is growing due to efforts by development partners and the private sector to create awareness. Access to information and training on HSTs has played a critical role in driving the demand at the household level. Strengthening formal and informal communication channels such as radio, extension services and “word of mouth” by farmers can help increase awareness of HSTs. Hermetic storage has imparted several benefits to farmers including food security, food safety and income. By storing grain in hermetic bags for 6 months or more, farmers can significantly increase their profits by taking advantage of seasonal price fluctuations, from post-harvest lows to pre-harvest peaks. Exploring finance mechanisms to ease cash constraints at harvest could further accelerate the adoption and use of HSTs among smallholder farmers. Additional efforts including investments to increase awareness and policies to streamline postharvest activities into government interventions would lead to sustained demand and adoption of hermetic technologies among smallholder farmers. Developing a private sector-led supply chain to increase availability in rural areas and adoption of HSTs will ensure sustained reduction in postharvest losses by smallholder farmers.

## CRediT authorship contribution statement

**D. Baributsa:** Conceptualization, Formal analysis, Funding acquisition, Methodology, Project administration, Resources, Supervision, Validation, Visualization, Software, Writing - original draft, Writing - review & editing. **A.W. Njoroge:** Data curation, Formal analysis, Investigation, Methodology, Supervision, Validation, Visualization, Software, Writing - original draft, Writing - review & editing.

## Declaration of competing interest

Dr. Dieudonne Baributsa is a co-founder of PICS Global Inc. a social enterprise that commercializes post-harvest technologies (including PICS bags) to smallholder farmers across the world and hence declares a conflict of interest.

Dr. Anastasia Njoroge declares no conflict of interest.

The funder had no role in the design of the study; in the collection, analyses, or interpretation of data; in the writing of the manuscript, or in the decision to publish the results.

.
